# Bistability in a system of two species interacting through mutualism as well as competition: Chemostat vs. Lotka-Volterra equations

**DOI:** 10.1371/journal.pone.0197462

**Published:** 2018-06-06

**Authors:** Stefan Vet, Sophie de Buyl, Karoline Faust, Jan Danckaert, Didier Gonze, Lendert Gelens

**Affiliations:** 1 Interuniversity Institute of Bioinformatics in Brussels (IB2), VUB-ULB, Brussels, Belgium; 2 Applied Physics Research Group, Vrije Universiteit Brussel (VUB), Brussels, Belgium; 3 Unité de Chronobiologie théorique, Université Libre de Bruxelles (ULB), Brussels, Belgium; 4 Laboratory of Molecular Bacteriology, KU Leuven, Leuven, Belgium; 5 Laboratory of Dynamics in Biological Systems, KU Leuven, Leuven, Belgium; University of Edinburgh, UNITED KINGDOM

## Abstract

We theoretically study the dynamics of two interacting microbial species in the chemostat. These species are competitors for a common resource, as well as mutualists due to cross-feeding. In line with previous studies (Assaneo, et al., 2013; Holland, et al., 2010; Iwata, et al., 2011), we demonstrate that this system has a rich repertoire of dynamical behavior, including bistability. Standard Lotka-Volterra equations are not capable to describe this particular system, as these account for only one type of interaction (mutualistic or competitive). We show here that the different steady state solutions can be well captured by an extended Lotka-Volterra model, which better describe the density-dependent interaction (mutualism at low density and competition at high density). This two-variable model provides a more intuitive description of the dynamical behavior than the chemostat equations.

## Introduction

Microorganisms form complex communities whose members compete for nutrients, cross-feed metabolites, parasitize each other and engage in a variety of other interactions. The recent advance of sequencing technology has greatly increased our knowledge of microbial community composition and in particular its change over time [4, 5]. Numerous studies have explored interactions within microbial communities and between hosts and their microorganisms [6–9], thereby showing that microorganisms compete with and parasitize each other, cross-feed and engage in all the other ecological interaction types known from macro-ecology (i.e. mutualism, commensalism, amensalism, competition and exploitation). These data sets form the basis for the development and parameterization of community models [10, 11], which in turn serve to deepen our understanding of community structure and dynamics (e.g. [12, 13]) and allow predicting the community response to perturbations such as change in diet [14] and pathogens [15].

The Lotka-Volterra equation is a classical way to model competition between species [16, 17] that has been generalized to multiple species engaging in all types of ecological interactions [18, 19]. Thanks to its ease of parameterization and well-established mathematical properties, as the interactions are modeled via linear functions in the growth rates, this generalized Lotka-Volterra (gLV) equation is frequently employed to model microbial communities [15, 20, 21]. Its simplicity enabled the formulation of a number of criteria to test for the stability of the coexistence equilibrium, where all species survive together [12, 22]. However, the gLV model provides a greatly simplified view on microbial communities, since it ignores the interaction mechanism, e.g. in the case of cross-feeding, the kinetics of the metabolite(s) that is/are exchanged between the species. In addition, it assumes that microbial community dynamics can be described by considering only pair-wise interactions and that effects of interacting species can be added (“additivity assumption”). The total fitness of a species is then the sum of the individual fitness with the fitness influences of the species it interacts with [23].

While recent experiments demonstrated that community dynamics can be predicted to a certain extent from pair-wise interactions [6, 24], the additivity assumption and the neglect of the interaction mechanism are serious limitations of the gLV. Recently, Momeni and colleagues have shown that some interaction mechanisms involving cross-feeding break the assumption of the gLV that the explicit modeling of interaction mediators can be neglected. For different interactions they compare numerically the mechanistic models, alternative pairwise models and LV models. They show that, depending on the interaction mechanism, pairwise models may fail [23]. Furthermore, there is experimental evidence where pair-wise interaction strengths do not add up in co-culture [25], while also theoretically the importance of higher order interactions, where there are multiple mediators, has been shown [26]. It is therefore still unclear to what extent the additivity assumption holds in microbial communities and in which conditions the gLV formalism is applicable.

Here, we want to address an interesting case, namely the simultaneous presence of a mutualistic and a competitive relationship between two species. Such a situation occurs when bacteria cross-feed essential co-factors, but in parallel compete for the main carbon source which can happen for instance in human gut microbiota (e.g. [27, 28]). If several species compete for a single nutrient, the competitive exclusion principle predicts that only a single species will survive in the long term [29]. In general, coexistence of species at steady state is only possible when the number of competing species is smaller than the number of different nutrients they compete for [30]. This principle inspired Hutchinson’s paradox of the plankton [31], which refers to the surprisingly large diversity of plankton species supported by a small number of nutrients. Several solutions to this paradox have been proposed. One suggested solution is to allow the species to coexist dynamically, e.g. in an oscillatory or chaotic state [32, 33]. The presence of spatial heterogeneity also permits the co-existence of several competing species (e.g. [34]). In addition, the competitive exclusion principle breaks down when including other types of interactions, such as mutualism [27] or cross-feeding [35]. Thus, even in a well-mixed environment, we expect to find scenarios where two competing and mutually cross-feeding species survive.

The LV equations are often employed to model pair-wise interactions [16, 36, 37]. However, it is challenging to model the presence of several interactions between two species with the LV formalism, since only the combined interaction strength can be represented. Our goal here is therefore to extend the LV equations in such a manner that the simultaneous occurrence of competition and mutualism can be accurately modeled.

To extend the LV model, we turn to chemostat equations [38–41], which describe species interactions while taking the interaction mechanism into account. Chemostat equations are based on controlled cultures of microorganisms, which allow measuring growth, metabolite consumption and production kinetics. In its simplest form, a chemostat is a well-stirred growth vessel with a continuous inflow of fresh medium and outflow of cell culture medium, such that the culture volume is kept constant. The chemostat is frequently employed to study ecological interactions such as competition [38]. In contrast to LV models, chemostat models explicitly take into account nutrient concentrations and can therefore capture more complex relationships, including the combination of mutualism and competition, thereby breaking the additivity assumption. Here, we show that the chemostat equations, which are relatively complex (five variables and nonlinear growth functions), can be simplified to a set of two extended LV equations. Every term has a simple, intuitive interpretation. The extended LV differs from the regular LV equations by including growth rates that are quadratic functions of the species abundances. We show that this reduced set of equations provides a good approximation of all steady-state solutions, and correctly captures the occurrence of bistability in a competitive-mutualistic system, as described previously [1, 42–44]. Moreover, as this reduced model is two-dimensional, we can illustrate its dynamics in the phase plane. This paper is organized in the following way. We start by introducing the chemostat system of two species that interact in a mutualistic and competitive manner and study its dynamical behavior. Next, we introduce the reduced model, a two-dimensional extended LV system. With this simplified model, we then analyze the dynamics in more detail, describe the conditions for bistability and discuss how well the extended LV captures the dynamics in the original chemostat system. We expect that a multispecies version of our extended model will better capture the dynamics of microbial communities than the generalized Lotka-Volterra model [4, 15, 20] as it allows to represent more complex dynamical behavior.

## Methods

We numerically simulate ordinary differential equations using *python* (Python Software Foundation. Python Language Reference, version 2.7. Available at http://www.python.org). More specifically, we use the integrate function of the package *scipy* [45].

In order to calculate a steady state over a range of parameters we use a Newton-Raphson method. This is an iterative method to find the optimal solution for the root of a set of equations, using the jacobian, see for example [46].

## Results

### Bistability in the chemostat system

**Fig 1:**
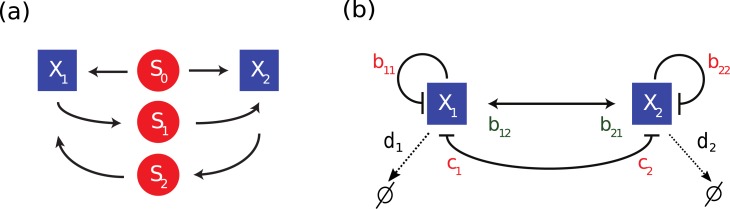
Schemes of the two-species system for the chemostat model (a) and the simplified model (b). Microbial species are represented by variables *X* (blue), nutrients by *S* (red), arrows represent the consumption or production of a nutrient by a species. In this system *X*_1_ and *X*_2_ compete for the consumption of *S*_0_ and they are mutualistic due to the cross-feeding through nutrients *S*_1_ and *S*_2_. Self-inhibition in the simplified system is determined via the parameter *b*_*ii*_ for species *X*_*i*_ (*i* = 1, 2), *b*_*ij*_ (*i* ≠ *j*) quantifies the strength of mutualism and *c*_*i*_ the strength of competition, *d*_*i*_ is the death rate.

The system we focus on consists of two microbial species (*X*_1_ and *X*_2_), which are competitors for a shared compound, the main carbon source *S*_0_ (Fig 1(a)). Additionally, they are also mutualists via cross-feeding of nutrients *S*_1_ and *S*_2_ (e.g. short-chain fatty acids such as formate and acetate). Without any such mutualism, the competition for the substrate *S*_0_ would lead to the extinction of one of the species, in agreement with the competitive exclusion principle. However, thanks to the mutualistic interaction, it is in principle possible that both species survive. In this model, an additional inflow of these cross-feeding nutrients is assumed, as this allows a species to survive without the other.

This system of bacteria (*X*_*i*_, *i* = 1, 2) and nutrients (*S*_*i*_, *i* = 0, 1, 2) can be modeled by a set of differential Eq (1) that are commonly used for the chemostat [38]:

$$\begin{array}{c} \begin{array}{cc} & \frac{dX_{1}}{dt}=\left(f_{1}\left(S_{0},S_{2}\right)-\Phi \right)X_{1},\\ & \frac{dX_{2}}{dt}=\left(f_{2}\left(S_{0},S_{1}\right)-\Phi \right)X_{2},\\ & \frac{dS_{0}}{dt}=\Phi \left({\tilde{S}}_{0}-S_{0}\right)+\nu_{01}f_{1}X_{1}+\nu_{02}f_{2}X_{2},\\ & \frac{dS_{1}}{dt}=\Phi \left({\tilde{S}}_{1}-S_{1}\right)+\nu_{11}f_{1}X_{1}+\nu_{12}f_{2}X_{2},\\ & \frac{dS_{2}}{dt}=\Phi \left({\tilde{S}}_{2}-S_{2}\right)+\nu_{21}f_{1}X_{1}+\nu_{22}f_{2}X_{2}, \end{array} \end{array}$$ (1)

where the meaning of the variables and parameters can be found in Table 1. Here, the parameters *ν* are defined positive for production, and negative for consumption of nutrients.

**Table 1:** Definition of the variables and parameters of the chemostat system Eq (1) and of the growth rates Eq (2).

Quantity	Symbol	Dimensions
Bacterial population density	*X*	$\frac{\text{number}}{\text{volume}}$
Nutrient concentration	*S*	$\frac{\text{mass}}{\text{volume}}$
Inflow of a nutrient	$\tilde{S}$	$\frac{\text{mass}}{\text{volume}}$
Growth rate of the bacteria	*f*	$\frac{1}{\text{time}}$
Flow rate	Φ	$\frac{1}{\text{time}}$
Production (consumption) constant	*ν*	$\frac{\text{mass}}{\text{number}}$
Maximal growth rate	*μ*	$\frac{1}{\text{time}}$
Half-saturation constant	*K*	$\frac{\text{mass}}{\text{volume}}$

The chemostat consists of a growth chamber with a continuous inflow and outflow. Thus, the flow rate Φ of fresh culture media and the input concentration of nutrients $\tilde{S_{i}}$ (*i* = 0, 1, 2) are tunable experimental parameters. The growth rates *f*_*i*_ (*i* = 1, 2) and the production and consumption constants *ν* are specific for every microbial species. We make the following assumptions:

- The death rate of the species is negligible in comparison with the flow rate.
- The production and consumption (determined by *ν*) of nutrients are proportional to the growth of the species.
- Growth rates are given by Eq (2), consisting of *μ*, the maximal growth rate, and Monod terms $\frac{S}{K+S}$, with *K* the half saturation constant:
  $$\begin{array}{c} \begin{array}{cc} & f_{1}\left(S_{0},S_{2}\right)=\mu_{1}\frac{S_{0}}{K_{10}+S_{0}}\frac{S_{2}}{K_{12}+S_{2}},\\ & f_{2}\left(S_{0},S_{1}\right)=\mu_{2}\frac{S_{0}}{K_{20}+S_{0}}\frac{S_{1}}{K_{21}+S_{1}}. \end{array} \end{array}$$ (2)
  These functions were introduced by Monod [47] and increase monotonically from zero to one with *S*. The Monod functions imply a strict dependence on both nutrients [27], such that a species requires both nutrients to grow.

This system has been described before, using different models, in [1–3], where bistability was observed as well. To assess how frequent bistability can occur, we performed 10.000 simulations using random parameter values. We find bistability in less than 0.1% of the cases. However, if we require the system to have a high inflow of *S*_0_, as well as a relatively low inflow of *S*_1_ and *S*_2_, then we find that two distinct stable states are more frequently found (6% percent of the cases) (see S1 Text for more details). These operating conditions are more likely to lead to bistability as larger *S*_0_ ensures the species to grow, thus producing more of *S*_1_ and *S*_2_, which again further promotes their growth. In other words, a high inflow of *S*_0_, combined with a low inflow of the nutrients *S*_1_ and *S*_2_ strengthens the positive feedback that exists between *X*_1_ and *X*_2_. Therefore, in this work, we assume such a high inflow of *S*_0_, combined with a low inflow of *S*_1_ and *S*_2_ (${\tilde{S}}_{1}$ and ${\tilde{S}}_{2}$ are small, but non-zero). This approach allows us to illustrate the widest range of the possible dynamics in this chemostat system, as in the absence of inflow of *S*_1_ and *S*_2_ a species can not survive by itself.

In what follows, we shortly summarize how the chemostat system behaves for different values of the growth rates ***μ***, using the parameter set (3), unless mentioned otherwise.

$$\begin{array}{c} \begin{array}{cc} & \phi =2,\\ & \mu =[\begin{array}{cc} \mu_{1} & \mu_{2} \end{array}]=[\begin{array}{cc} 1600 & 1600 \end{array}],\\ & \tilde{S}=[\begin{array}{ccc} {\tilde{S}}_{0} & {\tilde{S}}_{1} & {\tilde{S}}_{2} \end{array}]=[\begin{array}{ccc} 50 & 1 & 1 \end{array}],\\ & K=[\begin{array}{cc} K_{10} & K_{12}\\ K_{20} & K_{21} \end{array}]=[\begin{array}{cc} 200 & 200\\ 200 & 200 \end{array}],\\ & \nu =[\begin{array}{cc} \nu_{01} & \nu_{02}\\ \nu_{11} & \nu_{12}\\ \nu_{21} & \nu_{22} \end{array}]=[\begin{array}{cc} -1 & -1\\ 0.2 & -0.1\\ -0.1 & 0.2 \end{array}]. \end{array} \end{array}$$ (3)

**Fig 2:**
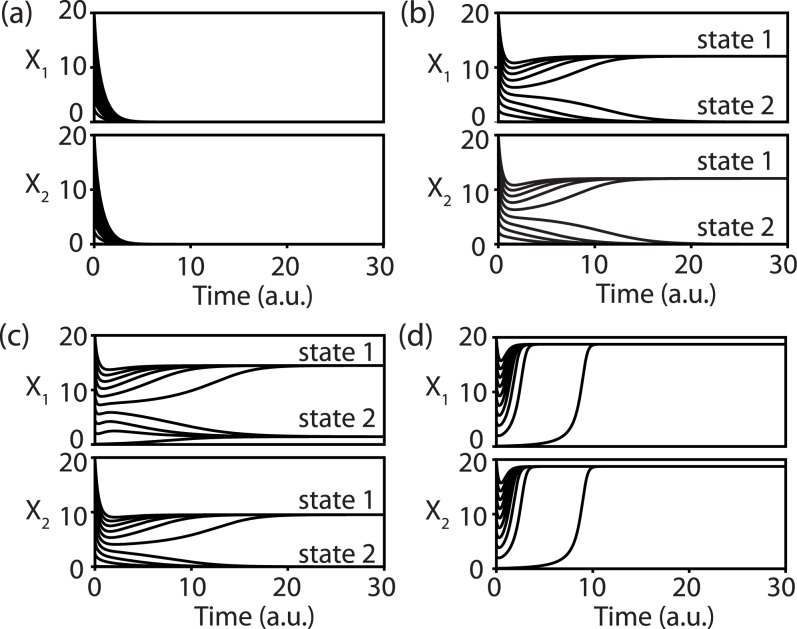
Time simulations for different values of the growth rates *μ* illustrate the different behavioral regimes of the system. For each panel 10 simulations are shown using initial densities varied between 0 and 20. (a) Extinction of the species *X*_1_ and *X*_2_ for all initial densities (***μ*** = [800, 800]). (b) Bistability: depending on the initial densities the species will either survive (state 1) or become extinct (state 2) (***μ*** = [1600, 1600]). (c) Bistability: There are two final states possible: coexistence of the species, *X*_1_ ≠ 0 and *X*_2_ ≠ 0 (state 1) or extinction of species *X*_2_ (state 2) (***μ*** = [2400, 1200]). (d) Survival of *X*_1_ and *X*_2_ for all initial densities (***μ*** = [2400, 2400]).

Varying the growth rates ***μ*** of the species, we find various regions of qualitatively different behavior (Fig 2). For low growth rates ***μ*** all species die out, regardless of the initial densities (Fig 2(a)). When both growth rates are increased, bistability exists, where the species coexist or die out, depending on the initial densities (Fig 2(b)). If the growth rates are not balanced, then it is also possible that one species survives or dies out, whereas the other one always survives, albeit with a different final density (Fig 2(c)). Finally, if both growth rates are large, then both species survive for all initial values (Fig 2(d)). In the next section, we introduce an extended LV-like model, with which we show and interpret the occurrence of these different types of behavior.

### Derivation of an extended Lotka-Volterra model

The consumption and production of compounds is explicitly modeled in the chemostat equations, making it a quantitative model that can be related to the actual experimental system. However, this also increases the complexity of the system. Our goal here is to reduce the chemostat model to a model of only two equations with reduced complexity, allowing for a qualitative dynamical analysis, while retaining a physical interpretation of each term in the model. This model reduction consists of the following critical steps:

1. Elimination of the nutrient variables, which is possible since linear relations between *S* and *X* exist for $t\gg \frac{1}{\Phi }$. This means, however, that the transient dynamics of the system is no longer accurately captured in the reduced system.
2. Simplification of the growth rates using a Taylor approximation when *K* ≫ *S*. It is always possible to find a parameter set for which the system has the same steady states that obeys this condition.
3. Elimination of higher order terms that are not critical for the qualitative dynamics.

For a more detailed discussion of this derivation and the corresponding approximations, see S2 Text. We obtain the following two equations:

$$\begin{array}{c} \begin{array}{cc} & \frac{dX_{1}}{dt}=(r_{1}\left(a_{1}-b_{11}X_{1}+b_{12}X_{2}-c_{1}X_{2}^{2}\right)-d)X_{1},\\ & \frac{dX_{2}}{dt}=(r_{2}\left(a_{2}-b_{22}X_{2}+b_{21}X_{1}-c_{2}X_{1}^{2}\right)-d)X_{2}, \end{array} \end{array}$$ (4)

where the relations of the new parameters with the chemostat parameters are listed here:

$$\begin{array}{c} \begin{array}{cc} & d=\Phi ,\\ & r_{1}=\frac{\mu_{1}}{K_{01}K_{21}},r_{2}=\frac{\mu_{2}}{K_{02}K_{12}},\\ & a_{1}={\tilde{S}}_{0}{\tilde{S}}_{2},a_{2}={\tilde{S}}_{0}{\tilde{S}}_{1},\\ & b_{11}=-\nu_{21}{\tilde{S}}_{0}-\nu_{01}{\tilde{S}}_{2},b_{22}=-\nu_{12}{\tilde{S}}_{0}-\nu_{02}{\tilde{S}}_{1},\\ & b_{12}=+\nu_{22}{\tilde{S}}_{0}+\nu_{02}{\tilde{S}}_{2},b_{21}=+\nu_{11}{\tilde{S}}_{0}+\nu_{01}{\tilde{S}}_{1},\\ & c_{1}=-\nu_{22}\nu_{02},c_{2}=-\nu_{11}\nu_{01}. \end{array} \end{array}$$ (5)

Here it can also be observed that if there is no external inflow of the cross-feeding nutrients *S*_*i*_ ($\tilde{S_{i}}=0$, *i* = 1, 2) this would limit the dynamic possibilities as *a*_*i*_ would be zero and the species would not be able to survive without their partner. The values corresponding to the parameter set (3) that was used in Fig 2 are now:

$$\begin{array}{c} \begin{array}{cc} & d=\Phi =2,\\ & r=[\begin{array}{cc} r_{1} & r_{2} \end{array}]=[\begin{array}{cc} 0.027 & 0.027 \end{array}],\\ & a=[\begin{array}{cc} a_{1} & a_{2} \end{array}]=[\begin{array}{cc} 50 & 50 \end{array}],\\ & b=[\begin{array}{cc} b_{11} & b_{12}\\ b_{21} & b_{22} \end{array}]=[\begin{array}{cc} 5 & 10\\ 10 & 5 \end{array}],\\ & c=[\begin{array}{cc} c_{1} & c_{2} \end{array}]=[\begin{array}{cc} 0.2 & 0.2 \end{array}]. \end{array} \end{array}$$ (6)

It is possible to renormalize the equations by dividing by the growth rates ***r*** to reduce the amount of parameters to 9. We choose not to do this to keep a clear connection with the chemostat equations. The concentration of input nutrients is modeled via the parameters ***a***. The parameters ***b*** quantify the linear interactions in the growth term, either due to self-limitation (carrying capacity), or due to the beneficial effect of the other species (mutualism). The quadratic term in the growth rates, ***c***, describes the competition between the species. This term is negligible for small densities of the competing species, but becomes more important when the densities increase. Finally, the parameter *d* characterizes the flow rate. In relation to the LV equations, *d* can also be seen as a death rate. The interactions can be represented in a simple scheme as in Fig 1(b). Our model distinguishes itself from the classical LV equations in that the growth rates are quadratic functions of the species densities. We will show that this additional nonlinearity allows for the bistable behavior of the system.

### Bistability in the extended Lotka-Volterra model

**Fig 3:**
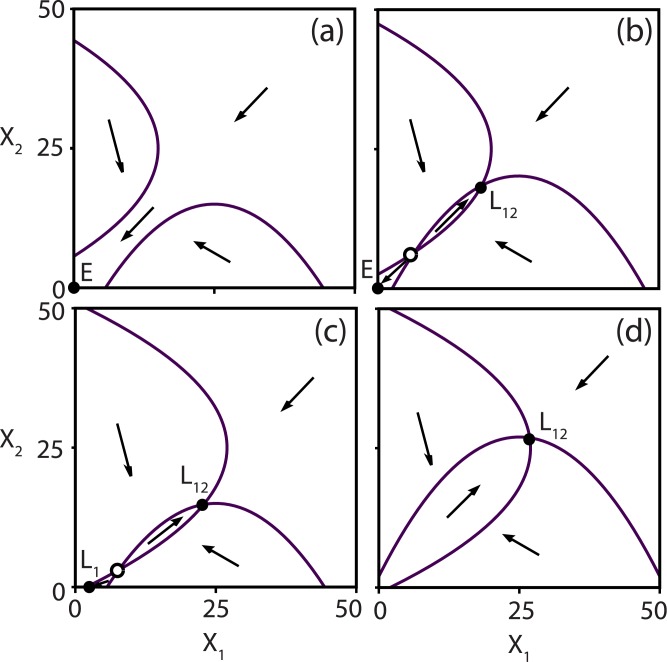
Phase plane (*X*_1_, *X*_2_) with nullclines for Eq (4) for increasing growth rates: *r* = [0.02, 0.02] (a), [0.027, 0.027] (b), [0.05, 0.02] (c), [0.05, 0.05](*d*). Different steady state configurations are found at the intersections of the nullclines: both species become extinct *E*, both species survive *L*_12_, only one species survives *L*_1_, *L*_2_. Stable solutions are indicated by the solid circle, while unstable saddle solutions are shown by the open circle.

In order to analyze the steady state solutions in the extended Lotka-Volterra model (4), we study the nullclines in the phase plane. The nullclines of the system are defined by $\frac{dX_{i}}{dt}=0$ (*i* = 1, 2), and these are either parabolae, determined by $a_{i}-b_{ii}X_{i}+b_{ij}X_{j}-c_{i}X_{j}^{2}=0$ when the species are alive (*X*_*i*_ > 0), or the *X*_1_- and/or *X*_2_-axis when the species are dead (*X*_*i*_ = 0). At the intersection of these nullclines, steady state solutions are found (see Fig 3), which we have given the following names:

- **E**: extinction of both species.
- **L**_1_: survival of species *X*_1_, extinction of species *X*_2_.
- **L**_2_: survival of species *X*_2_, extinction of species *X*_1_.
- **L**_12_: survival of both species.

Different situations are illustrated in Fig 3. If the parabola do not intersect, which happens for small growth rates, there is no state where both species can survive (Fig 3(a)). As a result both species become extinct (**E**) or only one species survives (**L**_1_ or **L**_2_). However, if the growth rates of one or both species are increased, a stable state **L**_12_ is created in a saddle-node bifurcation (SN, see S1 Fig). This leads to bistability between **E** and **L**_12_ (Fig 3(b)), or between **L**_*i*_ (*i* = 1, 2) and **L**_12_ when *r*_1_ ≠ *r*_2_ (Fig 3(c)). In each case, the boundary between the two basins of attractions is defined by the unstable manifold of the saddle point. When increasing the growth rates even further, the solutions **E** or **L**_*i*_ (*i* = 1, 2) lose their stability in a transcritical bifurcation (T, S1 Fig), such that only one stable fixed point remains, being **L**_12_ (Fig 3(d)).

**Fig 4:**
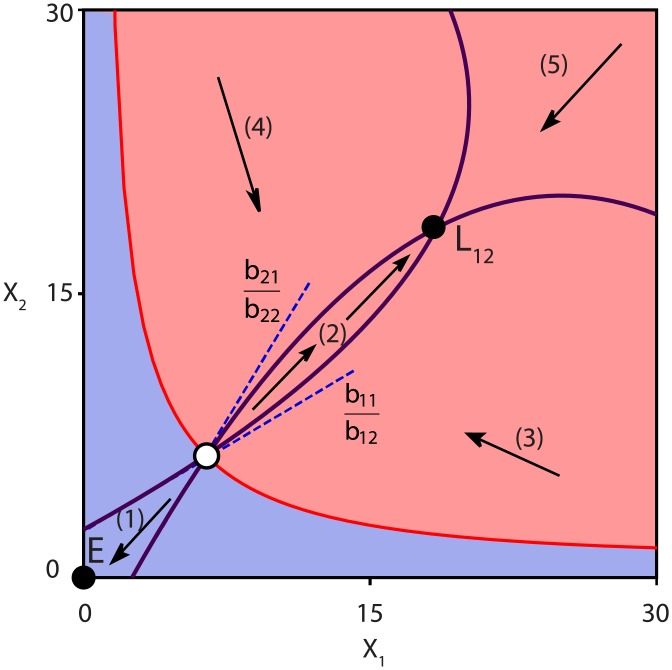
Zoom of Fig 3(b), using the same notation. The blue dashed lines are the linear approximations of the nullclines and need to intersect in order to have bistability. The regions (1)-(5) are discussed in the text. The blue and red areas correspond to the basins of attraction of each steady state.

In order to find criteria for bistability, we analyze the phase space depicted in Fig 3(b) in more detail. A zoom of this situation is shown in Fig 4. When the densities of *X*_1_ and *X*_2_ are both very low (region (1)), the linear and quadratic terms in the growth rates are negligible, such that the dynamics is governed by *r*_*i*_ *a*_*i*_ − *d*, which is negative here. Both species become extinct in this region because the species’ growth cannot compensate for the outflow. Therefore, *r*_*i*_ *a*_*i*_ − *d* < 0 is a first condition for bistability to occur. If only one of the species fullfils this condition, then the other one will always survive and the bistability is such as in Fig 3(c). If *r*_*i*_ *a*_*i*_ − *d* is too negative, then the nullclines will no longer intersect, leading to monostability of **E** (Fig 3(a)). Note that in the situation that there is no inflow of the cross-feeding nutrients (${\tilde{S}}_{i}=0$, for *i* = 1, 2), then *a*_*i*_ = 0 so that the first condition is always fulfilled. Therefore the system will be bistable for low flow rates, or both species go extinct for higher flow rates.

More generally, for small densities of the species the nullclines can be approximated by linear functions, hence *r*_*i*_(*a*_*i*_ − *b*_*ii*_ *X*_*i*_ + *b*_*ij*_ *X*_*j*_) − *d* = 0, these are the blue dashed lines in Fig 4. As the nullclines need to intersect for bistability, the slope of $\frac{dX_{2}}{dt}=0$ ($\frac{b_{21}}{b_{22}}$) needs to be larger than the slope of $\frac{dX_{1}}{dt}=0$ ($\frac{b_{11}}{b_{12}}$). Using these linear approximations, we obtain the constraint *b*_21_ *b*_12_ > *b*_11_ *b*_22_ as a second condition for bistability. In other words, this means that mutualism needs to be stronger than self-inhibition. In region (2) of Fig 4 the mutualism between the two species is dominant, leading to a positive growth for both species. However, in region (5) the quadratic terms become larger as the species densities are higher. Hence the growth is negative here due to the competition. The fixed point **L**_12_ is thus the result of a delicate balance between mutualism and competition. In regions (3) and (4) one density (e.g. *X*_1_) is large and the other smaller (e.g. *X*_2_), so that $\frac{dX_{1}}{dt}<0$ due to self-inhibition and $\frac{dX_{2}}{dt}>0$ due to mutualism. This will lead to either extinction or coexistence of the species, depending on the initial densities, as respectively indicated by the blue and red region in Fig 4.

In conclusion, there exist two necessary (but not sufficient) conditions for bistability:

1. *r*_1_ *a*_1_ − *d* < 0 and/or *r*_2_ *a*_2_ − *d* < 0
2. *b*_21_ *b*_12_ > *b*_11_ *b*_22_

Looking at the significance of these parameters in chemostat (Eq (5)), these conditions become:

1. $\frac{\mu_{1}}{K_{01}K_{21}}{\tilde{S}}_{0}{\tilde{S}}_{2}-\Phi <0$ and/or $\frac{\mu_{2}}{K_{02}K_{12}}{\tilde{S}}_{0}{\tilde{S}}_{1}-\Phi <0$
2. *ν*_11_ *ν*_22_ > *ν*_12_ *ν*_21_

Where we used ${\tilde{S}}_{i}⪡{\tilde{S}}_{0}$, for *i* = 1, 2, to obtain the second condition. The first condition means that the flow rate Φ needs to sufficiently large. The second condition means that the production of the cross-feeding nutrients needs to compensate for their consumption.

### Comparing the chemostat model with the extended Lotka-Volterra model

**Fig 5:**
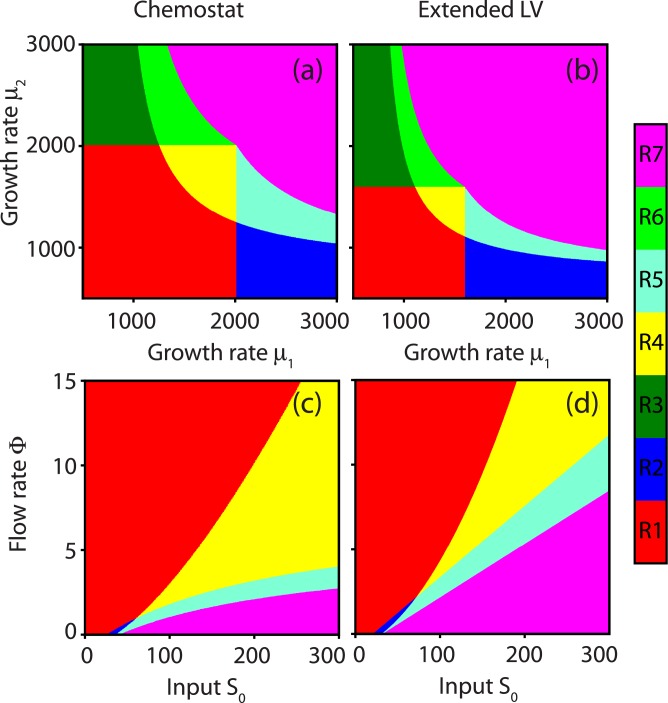
Different dynamical regimes for the mutualist-competitive system exist, depending on the values of the parameters. We define these regimes as follows: R1: Extinction. R2: Competitive exclusion, only species *X*_1_ survives. R3: Competitive exclusion, only species *X*_2_ survives. R4: bistability between extinction and coexistence. R5: bistability between survival of *X*_1_ and coexistence. R6: bistability between survival of *X*_2_ and coexistence. R7: coexistence of *X*_1_ and *X*_2_. (a) Influence of the growth rates ***μ*** in the chemostat system (b) Influence of the growth rates ***μ*** in the extended LV model (c) Influence of the flow rate Φ and the inflow ${\tilde{S}}_{0}$ in the chemostat for ***μ*** = [1600, 800]. (d) Influence of the flow rate Φ and the inflow ${\tilde{S}}_{0}$ in the extended LV model for ***r*** = [0.04, 0.02].

Next, we performed a bifurcation analysis to study how the stability of the fixed points changes as a function of the parameters (Fig 5). We did this for the chemostat Eq (1), as well as for its corresponding extended Lotka-Volterra model (4). The results for changing values of the growth rates are shown in Fig 5(a) for the chemostat model and in Fig 5(b) for the extended Lotka-Volterra model. Similarly, the influence of the experimental parameters Φ and ${\tilde{S}}_{0}$ are shown in Fig 5(c) and 5(d).

When changing the growth rates, the resulting behavior is very similar in both models (Fig 5(a) and 5(b)). There is extinction of one or both species for low growth rates (R1,R2,R3), corresponding to the situation illustrated in Fig 3(a). A slightly larger growth rate causes bistability (R4, R5 or R6), such as in Fig 3(b) and 3(c). Finally, when the growth rates are sufficiently large both species will always coexist (R7, Fig 3(d)).

Changing the experimental flow rates Φ and ${\tilde{S}}_{0}$ also reveals similar steady state dynamics of both models (Fig 5(a) and 5(b)). However, in the extended Lotka-Volterra model species are more likely to survive for higher values of ${\tilde{S}}_{0}$, as can be seen by the increased area of regions R2 and R7. Similarly, this is also highlighted by the smaller size of regions R1 and R4. This discrepancy between the chemostat and the extended LV model is mainly explained by the neglection of the higher order terms in the simplification. Thus, when quantitative results are required, including these terms would lead to a better correspondence between both models. In general, the flow rate Φ needs to be low in comparison to the inflow ${\tilde{S}}_{0}$ in order to allow for survival of the species. Bistability will not occur if the flow rate is too low or the inflow ${\tilde{S}}_{0}$ too high.

Although both models show very similar behavior of their steady-state solutions, the extended Lotka-Volterra is highly simplified and therefore has its limitations. The agreement between the chemostat equations and the extended Lotka-Volterra model is lost when the parabola intersect more than twice (see S2 Fig). Although two stable fixed points of coexistence are predicted by the intersecting parabolic nullclines, we did not observe this behavior in the chemostat equations. This type of behavior can occur when the maximum of $\frac{dX_{1}}{dt}=0$ is situated below and to the right of the maximum of $\frac{dX_{2}}{dt}=0$. Using the values of the maxima of parabola we find that this does not happen when the following conditions are satisfied:

$$\begin{array}{c} \begin{array}{cc} & \frac{b_{21}}{2c_{2}}>\frac{\frac{b_{12}^{2}}{4c_{1}}-(\frac{d}{r_{1}}-a_{1})}{b_{11}},\\ & \frac{b_{12}}{2c_{1}}>\frac{\frac{b_{21}^{2}}{4c_{2}}-(\frac{d}{r_{2}}-a_{2})}{b_{22}}. \end{array} \end{array}$$ (7)

A physical interpretation of these conditions is not straightforward. However, one can see that the parameters for self-inhibition *b*_11_ and *b*_22_ should be sufficiently large to balance the parameters for the mutualistic strength *b*_12_ and *b*_21_.

## Discussion

In this theoretical study we discussed the dynamics of two interacting microbial species in the chemostat. Both species compete for a common resource, while also being mutualists through cross-feeding. Regions of bistable behavior were found in this five-dimensional chemostat system through random parameter selection. In order to gain more insight into the origin of the bistability, we derived an extended Lotka-Volterra (LV) model. As this model is two-dimensional, it allowed us to study the system dynamics in the phase plane using nullclines. Our model differs from the classical LV system in that it has a quadratic term modeling the competition, while the typical linear term describes the mutualistic interaction. All parameters in our model have a clear relation to the original chemostat parameters, such as the inflow of nutrients and outflow of biomass.

We showed that the steady state solutions are qualitatively very similar in both models, such that our model can be effectively used to study such steady-state dynamics. We do not expect temporal dynamics to be accurately captured by this system as it is only a good approximation of the chemostat system after some initial transient time. Using the extended LV system, we analyzed various regions of bistable behavior and its dependence on system parameters. While neglecting higher order terms led to larger regions in parameter space where both species survive, we found good qualitative agreement between both models. We showed that bistability occurs when the mutual dependence on the cross-feeding nutrients is sufficiently high, which is the case when the system has a low inflow of the cross-feeding nutrients and a relatively high inflow of *S*_0_. For small microbial densities, one or both species will be washed out if one or both growth rates are not sufficient to compensate for the flow rate. However, for larger densities, the growth rate is increased due to mutualism until the densities reach a point where competition limits the growth and the two species can coexist. This creates bistability between one state that has insufficient growth rate and another state where there is a balance between mutualism and competition.

The existence of bistability in such systems has been shown before using different models [1–3]. On the one hand bistability was analyzed more generally using a phase plane analysis [2, 3], while on the other hand chemostat models were also used where mutualism was incorporated in a general function in the species equations [1]. Our approach distinguishes itself from these studies in that we simplify chemostat equations to a two-variable model in which all parameters retain a clear relation to the original chemostat parameters. The advantage of such simplified model is that the dynamics of the system can be analyzed and visualized in the phase plane. For example, by analyzing how the shape of nullclines depend on the different competitive and mutualistic interaction parameters, the existence of bistability can be easily found. Interpreting the existence of bistability in the set of five chemostat equations is considerably more challenging and less intuitive as its description is often limited to numerical simulations.

Our findings are not only limited to chemostat systems, but are expected to be relevant for any system with competition and mutualism. For example, plants release carbon that allows the development of microbes in the rizosphere [48]. These microbes can interact with each other in many different ways, such as through mutualism [7]. Two mutualistic species that both grow on the released carbon could be described by our extended LV model. Furthermore, this model could likely be generalized to model the behavior of more than two interacting microbes. How such generalized systems allow for the coexistence of multiple stable states is a question for future research. We believe that such extensions of the generalized LV equations are an important step towards developing microbial community models that are both more flexible and realistic. Additional realism could be introduced by considering growth not to be proportional to nutrient production, and examining how this would alter the derived extended LV equations. Finally, it is interesting to ask why cooperation exists within microbes despite the risks of free-riders. Certainly in the field of game theory, the influence of cheating behavior of individuals in a cooperating population has been widely studied [49–51]. A model like the extended LV could be another approach to predicting whether a population can survive in the presence of cheating. Cheaters would grow selfishly without producing the cross-feeding nutrients, resulting in lower values for the mutualistic parameters *b*_12_ and *b*_21_ and shifted nullclines, making it less likely for the species to coexist. In the phase plane (Fig 3) it seems that the overall population has a larger benefit by coexisting than by cheating, as the steady state concentration of coexistence is higher in all cases. It would be interesting to further explore how cheating behavior affects the dynamics of this system.

## Supporting information

S1 Fig Bifurcation diagram showing the density of the fixed points *X*_1_, *X*_2_ for increasing growth rates of the species *r*, keeping *r*_1_ = *r*_2_. Notation is as in Fig 3. A saddle-node bifurcation is indicated by SN and a transcritical bifurcation by T. Stable (unstable) solutions are plotted in a solid blue (dashed red) line.
(EPS)

S2 Fig Illustration of a situation where the similarity between chemostat equations and the toy model breaks down. The parabola intersect more than twice, so that two different stable fixed points exist where the species coexist. Parameters: *b*_11_ = *b*_22_ = 2, ***r*** = [0.023, 0.023].
(EPS)

S1 Text Random parameter simulations shows how frequent the system behaves bistable. (PDF)

S2 Text Derivation of the extended Lotka-Volterra equations. (PDF)

S3 Text Python script that creates time traces and a phase diagram for the extended LV and the chemostat model. This script shows how the extended LV and the chemostat model can be simulated using Python code.
(PY) Click here for additional data file.
