# Supplementary material for: Bistability in a system of two species interacting through mutualism as well as competition: Chemostat vs. Lotka-Volterra equations
Source: PLoS One. 2018 Jun 6;13(6):e0197462. doi: 10.1371/journal.pone.0197462 (PMC5991418; doi:10.1371/journal.pone.0197462)
Supplement: S1 Text — (PDF) [file pone.0197462.s003.pdf]

Random parameter simulations shows how frequent the system behaves bistable. We did simulations with random sets of parameters for the chemostat system to find how frequently bistability occurs. The simulations were performed in python, using the random module in the package *numpy*, fixing the seed at 62. The results are summarized in Fig.1. First we drew all parameters from a uniform random distribution between 0.1 and 100. For 10000 simulations we found bistability for 0.063% of the cases (Fig.1 (a)). Increasing all input concentrations did not lead to a better result, however, the result was affected when we increased only $\tilde{S}_0$ and not the cross-feeding nutrients. $\tilde{S}_0$ was here taken from a uniform distribution between 5 and 5000, leading to larger values of $\tilde{S}_0$ than of $\tilde{S}_1$ and $\tilde{S}_2$. We found that bistability occured with a percentage of 6.02% for 10000 simulations (Fig.1 (b)).

Therefore we conclude that the ratio of $\tilde{S}_0$ to $\tilde{S}_1$ and $\tilde{S}_2$ plays an important role in the possible existence of bistability. The species need to be sufficiently dependent on each others production of $\tilde{S}_1$ and $\tilde{S}_2$ in order for the system to be bistable.

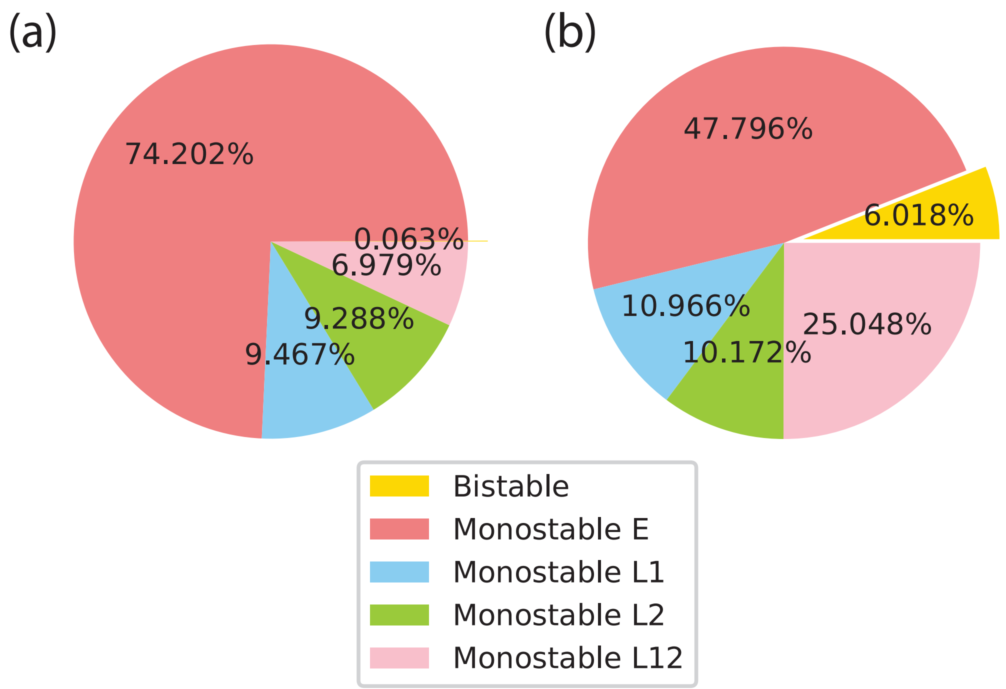

Fig 1. (Color online) (a) Pie chart for 10000 simulations with random parameters, where all parameters are taken from a uniform random distribution between 0.1 and 100. (b) Pie chart for 10000 simulations with random parameters, where $\tilde{S}_0$ is taken a from uniform random distribution between 5 and 5000, and the other parameters from a uniform random distribution between 0.1 and 100. The notation for the fixed points as follows: **E**: extinction of both species. $\mathbf{L}_1$: survival of species X_1, extinction of species X_2. $\mathbf{L}_2$: survival of species X_2, extinction of species X_1. $\mathbf{L}_{12}$: survival of both species.
