# Supplementary material for: Bistability in a system of two species interacting through mutualism as well as competition: Chemostat vs. Lotka-Volterra equations
Source: PLoS One. 2018 Jun 6;13(6):e0197462. doi: 10.1371/journal.pone.0197462 (PMC5991418; doi:10.1371/journal.pone.0197462)
Supplement: S2 Text — (PDF) [file pone.0197462.s004.pdf]

Detailed calculations to derive the extended Lotka-Volterra model. The derivation of the extended Lotka-Volterra model from the chemostat equations is carried out in three steps. First, we eliminate the nutrients from the equations to obtain a two-dimensional system. Secondly, we further simplify these equations using a Taylor approximation. This leads to growth rates that are second degree polynomial functions of the species concentrations. Finally, a last simplification consists in omitting two of the nonlinear terms of the growth rate.

The chemostat equations are given by:

$$\begin{aligned}
\frac{dX_1}{dt} &= (f_1(S_0, S_2) - \Phi)X_1, \\
\frac{dX_2}{dt} &= (f_2(S_0, S_1) - \Phi)X_2, \\
\frac{dS_0}{dt} &= \Phi(\tilde{S}_0 - S_0) + \nu_{01} f_1 X_1 + \nu_{02} f_2 X_2, \\
\frac{dS_1}{dt} &= \Phi(\tilde{S}_1 - S_1) + \nu_{11} f_1 X_1 + \nu_{12} f_2 X_2, \\
\frac{dS_2}{dt} &= \Phi(\tilde{S}_2 - S_2) + \nu_{21} f_1 X_1 + \nu_{22} f_2 X_2,
\end{aligned} \tag{1}$$

With growth rates:

$$\begin{aligned}
f_1(S_0, S_2) &= \mu_1 \frac{S_0}{K_{10} + S_0} \frac{S_2}{K_{12} + S_2}, \\
f_2(S_0, S_1) &= \mu_2 \frac{S_0}{K_{20} + S_0} \frac{S_1}{K_{21} + S_1}.
\end{aligned} \tag{2}$$

To eliminate the nutrients in the differential equations (1), we use a simplification following Ref. [1]. The following variables P_i are introduced:

$$\begin{aligned}
P_0 &= S_0 - \nu_{01} X_1 - \nu_{02} X_2, \\
P_1 &= S_1 - \nu_{11} X_1 - \nu_{12} X_2, \\
P_2 &= S_2 - \nu_{21} X_1 - \nu_{22} X_2.
\end{aligned} \tag{3}$$

These obey the differential equations $\frac{dP_i}{dt} = \Phi(\tilde{S}_i - P_i)$ $(i = 0, 1, 2)$ for which the solutions are given by exponentially decreasing functions $P_i(t) = \tilde{S}_i + (P_i(0) - \tilde{S}_i)e^{-\Phi t}$. After a transient time, when $t \gg \frac{1}{\Phi}$, the equations reduce to $P_i(t) = \tilde{S}_i$. Hence, we obtain the relations given in Eq.(4).

$$\begin{aligned}
\tilde{S}_0 &= S_0 - \nu_{01} X_1 - \nu_{02} X_2, \\
\tilde{S}_1 &= S_1 - \nu_{11} X_1 - \nu_{12} X_2, \\
\tilde{S}_2 &= S_2 - \nu_{21} X_1 - \nu_{22} X_2.
\end{aligned} \tag{4}$$

We can use these equations to eliminate S_0, S_1 and S_2 from the chemostat equations (Eq.(1)), and obtain a 2 dimensional system that only depends on the species concentrations (Eq.(5)).

$$\begin{aligned}
\frac{dX_1}{dt} &= (f_1(X_1, X_2) - \Phi)X_1, \\
\frac{dX_2}{dt} &= (f_2(X_1, X_2) - \Phi)X_2.
\end{aligned} \tag{5}$$

This system has a different transient behavior than the original chemostat system, but it converges to the same steady-state attractor as the original system. Indeed, the variables P_i are decreasing exponentially with time constant $\frac{1}{\Phi}$, implying the same qualitative dynamics in both systems for $t \gg \frac{1}{\Phi}$.

However, as the nutrient concentrations cannot be negative this gives some constraints for the concentrations in order to be realistic. When imposing $S_i > 0$ in Eq.4, we obtain the restrictions Eq.(6):

$$\begin{aligned} X_2 &< \frac{\tilde{S}_0 - \nu_{01}X_1}{\nu_{02}}, \\ X_2 &< \frac{-\tilde{S}_1 - \nu_{11}X_1}{\nu_{12}}, \\ X_2 &> \frac{+\tilde{S}_2 - \nu_{21}X_1}{\nu_{22}}. \end{aligned} \tag{6}$$

Using (4) to eliminate the nutrients S_i $(i = 0, 1, 2)$ in the growth rates (2), it is clear that the growth rates are highly nonlinear functions and difficult to interpret. Looking at the behavior of the species in extreme situations, for example when the nutrient concentrations are very low or very high, gives important insights about the dynamics of this system.

The Monod terms $S/(K + S)$ increase from zero to one with S. For high nutrient concentrations, $S \gg K$, this term approximates one, whereas for low concentrations, $S \ll K$, it behaves as a linear function S/K. Both growth rates in the chemostat system (2) depend on two nutrients, hence we can consider four different limiting situations $(i, j = 1, 2$ & $i \neq j)$:

- $K_{0i} \ll S_0$ & $K_{ji} \ll S_j$,
- $K_{0i} \gg S_0$ & $K_{ji} \ll S_j$,
- $K_{0i} \ll S_0$ & $K_{ji} \gg S_j$,
- $K_{0i} \gg S_0$ & $K_{ji} \gg S_j$.

We show that in the first three cases we obtain equations that are normal growth equations, similar to LV systems. The last situation is the most interesting case to obtain a system with the same dynamics as the chemostat system.

(a) $K_{0i} \ll S_0$ & $K_{ji} \ll S_j$. This is the simplest case. We obtain exponential growth equations Eq.(7) for both species since there are enough nutrients available. They do not depend on each other in a competitive nor mutualistic manner. As long as the growth rate is larger than the flow rate the populations increase exponentially. This ends when the consumption of nutrients has increased so much that the condition of high nutrient concentrations is no longer valid.

$$\begin{aligned} \frac{dX_1}{dt} &= (\mu_1 - \Phi)X_1, \\ \frac{dX_2}{dt} &= (\mu_2 - \Phi)X_2. \end{aligned} \tag{7}$$

(b) $K_{0i} \gg S_0$ & $K_{ji} \ll S_j$. Due to the relations Eq.(4) the growth rates reduce to linear functions of the species concentrations:

$$\frac{dX_1}{dt} = (\frac{\mu_1}{K_{01}}(\tilde{S}_0 + \nu_{01}X_1 + \nu_{02}X_2) - \Phi)X_1,$$
$$\frac{dX_2}{dt} = (\frac{\mu_2}{K_{02}}(\tilde{S}_0 + \nu_{01}X_1 + \nu_{02}X_2) - \Phi)X_2. \tag{8}$$

This is a competitive Lotka-Volterra system. The growth is limited by the compound S_0 in this case, so that the competition for S_0 dominates the system. Only one species survives due to competitive exclusion.

(c) $K_{0i} \ll S_0$ & $K_{ji} \gg S_j$. The growth rates are linear functions as well. However, here the growth is limited by the cross-feeding nutrients S_1 and S_2, so that we obtain mutualistic Lotka-Volterra equations:

$$\frac{dX_1}{dt} = (\frac{\mu_1}{K_{21}}(\tilde{S}_2 + \nu_{21}X_1 + \nu_{22}X_2) - \Phi)X_1,$$
$$\frac{dX_2}{dt} = (\frac{\mu_2}{K_{12}}(\tilde{S}_1 + \nu_{11}X_1 + \nu_{12}X_2) - \Phi)X_2. \tag{9}$$

Here, the concentrations keep increasing until the condition $K_{0i} \ll S_0$ is no longer valid and the competition for S_0 becomes significant.

(d) $K_{0i} \gg S_0$ & $K_{ji} \gg S_j$. The growth is limited by S_0, as well as by S_1 and S_2. Therefore both competition and mutualism play a significant role. The growth rates are now given by:

$$f_1 = r_1(\tilde{S}_0 + \nu_{01}X_1 + \nu_{02}X_2)(\tilde{S}_2 + \nu_{21}X_1 + \nu_{22}X_2),$$
$$f_2 = r_2(\tilde{S}_0 + \nu_{01}X_1 + \nu_{02}X_2)(\tilde{S}_1 + \nu_{11}X_1 + \nu_{12}X_2), \tag{10}$$

with $r_i = \mu_i/(K_{0i}K_{ji})$. As we are interested in explaining bistability in the chemostat system, where competition as well as mutualism are important, we use these equations to simplify the system.

Theoretically, the simplification is valid if the conditions $K_{0i} \gg S_0$ & $K_{ji} \gg S_j$ are fulfilled. However, if this is not the case, it is possible to find a parameter set with higher values of K for which the system has the same steady states. To understand this, we consider the steady state equations $\frac{dX_i}{dt} = 0$ and $\frac{dS_i}{dt} = 0$ of Eq.(1). It is possible to use a different parameter set to obtain the same solutions for the steady state concentrations of X_i and S_i. Such a change will make the dynamics faster or slower, but as the system converges to the same fixed points, it is the same qualitative behavior.

$$\frac{dX_i}{dt} = \mu_i \frac{S_0}{K_{0i} + S_0} \frac{S_j}{K_{ji} + S_j} - \Phi = 0 \tag{11}$$

Therefore, as long as Eq.(11) still yields the solutions for S_0 and S_j, we can change the parameters $\boldsymbol{\mu}$, Φ and K such that we find values for K for which $K_{0i} \gg S_0$ and $K_{ji} \gg S_j$. We show this with an example in Fig.1. In Fig.1(a) we show the dynamics simulated with parameter set as defined in the main text, with $\boldsymbol{\mu} = \begin{bmatrix}1600 & 1600\end{bmatrix}$, which leads to a bistable situation. As $K_{0i} = 200$ the conditions $K_{0i} \gg S_0$ are not satisfied. In Fig.1(b) the values of K are increased, so that $K' = 100K$, $\boldsymbol{\mu}$ is then changed according to Eq.(12), which follows from Eq.(11). Therefore the steady state concentrations remain the same, as can be seen in Fig.1(b).

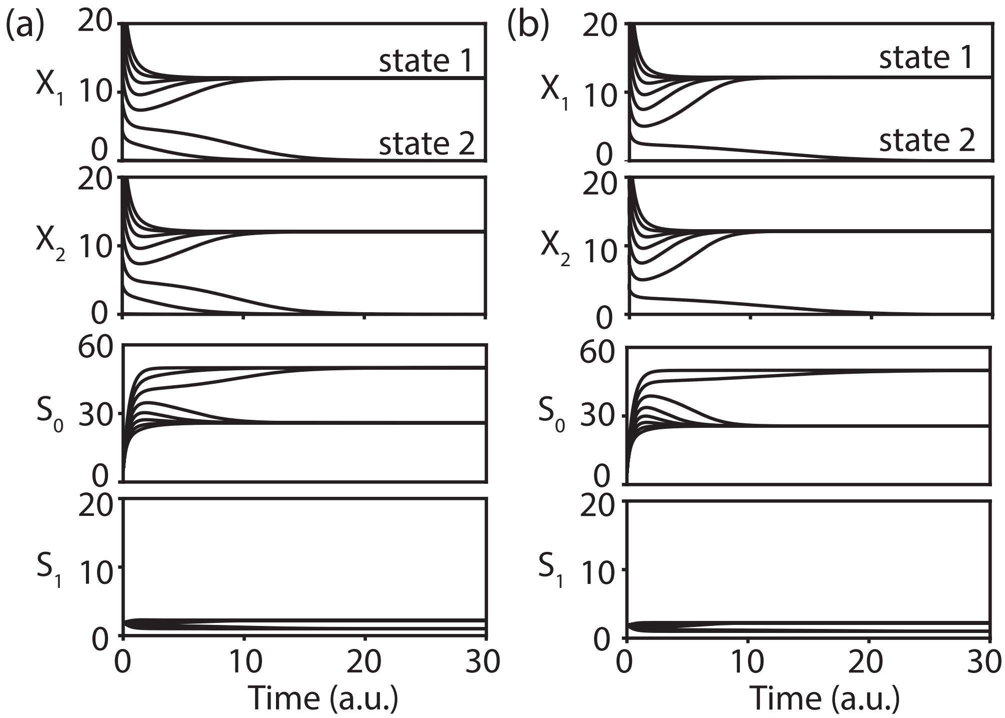

Fig 1. (a) Time simulations for 10 different initial densities of the species, varied between 0 and 20 of the chemostat system with $\boldsymbol{\mu} = [1600, 1600]$, $K_{0i} = 200$ and $K_{ij} = 200$. The species X_1 and X_2 either coexist (state 1) or die out (state 2). The end concentration of S_0 is $S_0 = \tilde{S}_0 = 50$ or $S_0 = 26$. Therefore the condition $S_0 \ll K_{0i}$ is not satisfied. The end concentrations of S_1, equal to those of S_2, are very small so that $S_i \ll K_{ij}$ is satisfied. (b) If we increase the values K such that $K' = 100K$ and we change $\boldsymbol{\mu}$ so that Eq.(12) is satisfied, then the qualitative dynamics is not changed. The steady state concentrations are the same as in (a), but the transient dynamics is (moderately) different. The condition $S_0 \ll K_{0i}$ is well satisfied for this parameter set, so that the approximation to Eq.(13) is valid.

$$\mu_i' \frac{S_0}{K_{0i}' + S_0} \frac{S_j}{K_{ji}' + S_j} = \mu_i \frac{S_0}{K_{0i} + S_0} \frac{S_j}{K_{ji} + S_j}, \tag{12}$$

By substituting the growth rates Eq.(10) in Eq.(5), we obtain the following system (with $i, j = 1, 2$ and $i \neq j$):

$$\frac{dX_i}{dt} = r_i(a_i - b_{ii}X_i + b_{ij}X_j - c_iX_j^2 + f_{ii}X_i^2 + f_{ij}X_iX_j - d)X_i \tag{13}$$

The relationships between these new parameters and those of the chemostat are

given by:

$$
\begin{aligned}
r_i &= \frac{\mu_i}{K_{i0}K_{ij}} \\
b_{ii} &= -\nu_{0i}\tilde{S}_j - \nu_{ji}\tilde{S}_0 \\
b_{ij} &= +\nu_{jj}\tilde{S}_0 + \nu_{0j}\tilde{S}_j \\
c_i &= -\nu_{0j}\nu_{jj} \\
f_{ii} &= \nu_{0i}\nu_{ji} \\
f_{ij} &= \nu_{0j}\nu_{ji} + \nu_{0i}\nu_{jj} \\
d &= \Phi
\end{aligned} \tag{14}
$$

The dynamics of system Eq.(13) can be visualized in the phase plane. As an example we did this for the situation where the behavior is bistable (Fig.2). The nullclines of the system are hyperbolic functions (as well as the x- and y-axis). The realistic region of the phase plane is limited by the restrictions given by Eq.(6), hence the only physical regime is situated inside the red lines in Fig.2. To further simplify the equations, we examine the effect of the different nonlinear terms in the growth: $c_{ij}X_j^2$, $f_{ii}X_i^2$ and $f_{ij}X_iX_j$. All these nonlinear terms become particularly relevant when the concentrations are large. In the realistic regime, this means that the growth is negative for large concentrations, which is caused by the competition. This is modeled via the term $c_{ij}X_j^2$, which is therefore necessary for the dynamics. The term $f_{ii}X_i^2$ causes the divergence in the growth when the branches of the hyperbola in the unrealistic regime are crossed, however they do not affect the qualitative behavior in the realistic regime. The last term $f_{ij}X_iX_j$ has the effect of increased competition or self-inhibition at high concentrations, where the growth already is negative. Therefore, the qualitative dynamics in the physical regime is not affected by elimination of the two terms $f_{ii}X_i^2$ and $f_{ij}X_iX_j$, as the competition at high concentrations is incorporated in the term $c_{ij}X_j^2$. The minimal model we use to describe the dynamics in the physical regime is therefore given by Eq.(15), which has parabolic nullclines. In this article, we refer to these equations as the extended Lotka-Volterra model.

$$
\begin{aligned}
\frac{dX_1}{dt} &= r_1(a_1 - b_{11}X_1 + b_{12}X_2 - c_1X_2^2)X_1 - dX_1 \\
\frac{dX_2}{dt} &= r_2(a_2 + b_{21}X_1 - b_{22}X_2 - c_2X_1^2)X_2 - dX_2
\end{aligned} \tag{15}
$$

References

1. Smith HL, Waltman P. The Theory of the Chemostat: Dynamics of Microbial Competition. Cambridge University Press; 1995.

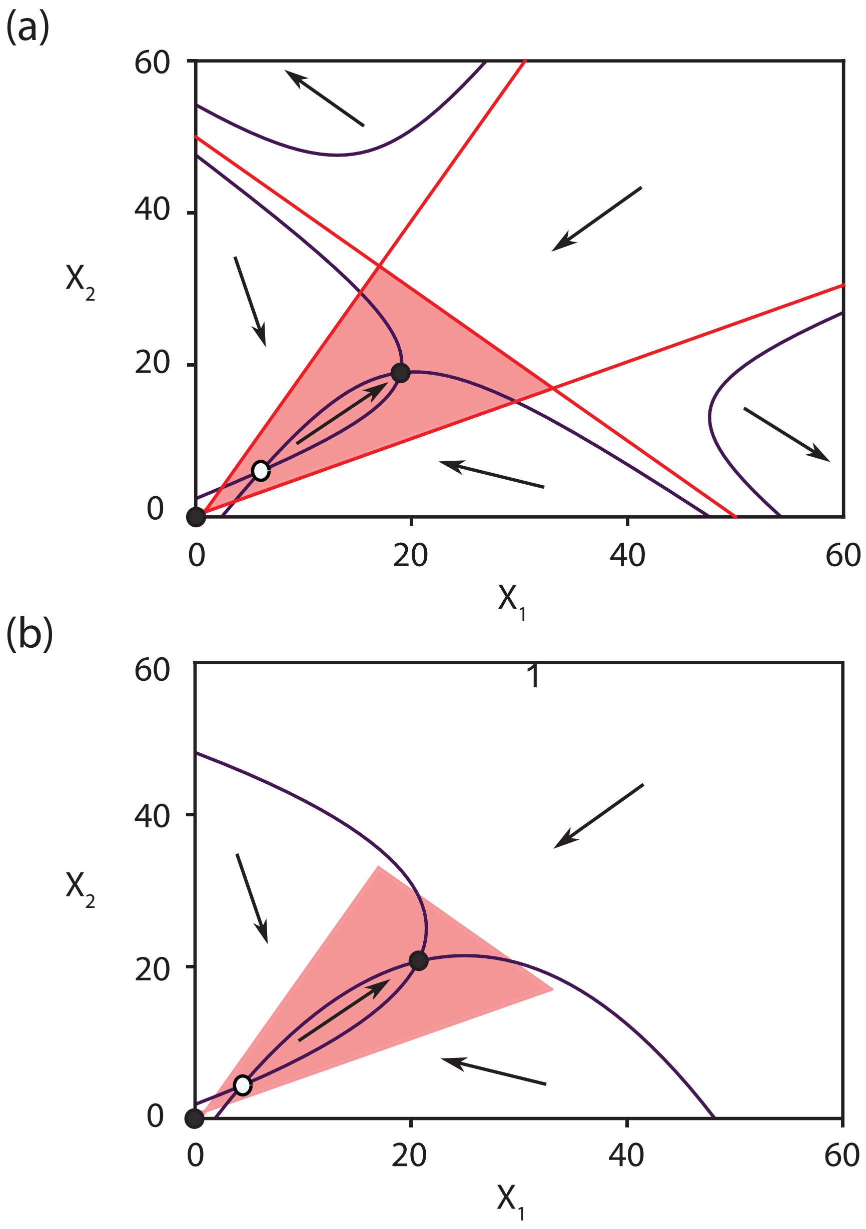

Fig 2. (Color online) (a) Phase plane of the system defined by Eq.(13). The nullclines are hyperbola and the x- and y-axis. The physical regime is restricted by Eq.(6), which is the region within the red lines colored in red. (Parameters: $\tilde{\boldsymbol{S}} = [50, 0.1, 0.1]$, $\boldsymbol{\mu} = [3500, 3500]$) (b) The reduced model as defined by Eq.(13) has parabolic nullclines and has the same qualitative dynamics as Eq.(13) in the physical regime (colored in red). (Parameters: $\tilde{\boldsymbol{S}} = [50, 0.1, 0.1]$, $\boldsymbol{r} = [0.087, 0.087]$)
